# Genetic Diversity and Population Structure in *Vicia faba* L. Landraces and Wild Related Species Assessed by Nuclear SSRs

**DOI:** 10.1371/journal.pone.0154801

**Published:** 2016-05-11

**Authors:** Hugo R. Oliveira, Diana Tomás, Manuela Silva, Susana Lopes, Wanda Viegas, Maria Manuela Veloso

**Affiliations:** 1 Plant Biology/CIBIO-Centro de Investigação em Biodiversidade e Recursos Genéticos, Universidade do Porto, Campus Agrário de Vairão, 4485–661, Vairão, Portugal; 2 Linking Landscape, Environment, Agriculture and Food (LEAF), Instituto Superior de Agronomia, Universidade de Lisboa, Tapada da Ajuda 1349–017, Lisboa, Portugal; 3 CTM.CIBIO-Centro de Investigação em Biodiversidade e Recursos Genéticos, Universidade do Porto, Campus Agrário de Vairão, 4485–661, Vairão, Portugal; 4 Unidade de Investigação de Biotecnologia e Recursos Genéticos, INIAV, Quinta do Marquês, 2784–505, Oeiras, Portugal; Institute for Sustainable Agriculture (IAS-CSIC), SPAIN

## Abstract

Faba bean (*Vicia faba* L.) is a facultative cross-pollinating legume crop with a great importance for food and feed due to its high protein content as well as the important role in soil fertility and nitrogen fixation. In this work we evaluated genetic diversity and population structure of faba bean accessions from the Western Mediterranean basin and wild related species. For that purpose we screened 53 *V*. *faba*, 2 *V*. *johannis* and 7 *V*. *narbonensis* accessions from Portugal, Spain and Morocco with 28 faba bean Single Sequence Repeats (SSR). SSR genotyping showed that the number of alleles detected per locus for the polymorphic markers ranged between 2 and 10, with Polymorphic Information Content (PIC) values between 0.662 and 0.071, and heterozygosity (H_O_) between 0–0.467. Heterozygosity and inbreeding coefficient levels indicate a higher level of inbreeding in wild related species than in cultivated *Vicia*. The analysis of molecular variance (AMOVA) showed a superior genetic diversity within accessions than between accessions even from distant regions. These results are in accordance to population structure analysis showing that individuals from the same accession can be genetically more similar to individuals from far away accessions, than from individuals from the same accession. In all three levels of analysis (whole panel of cultivated and wild accessions, cultivated faba bean accessions and Portuguese accessions) no population structure was observed based on geography or climatic factors. Differences between *V*. *narbonensis* and *V*. *johannis* are undetectable although these wild taxa are clearly distinct from *V*. *faba* accessions. Thus, a limited gene flow occurred between cultivated accessions and wild relatives. Contrastingly, the lack of population structure seems to indicate a high degree of gene flow between *V*. *faba* accessions, possibly explained by the partially allogamous habit in association with frequent seed exchange/introduction.

## Introduction

Faba bean (*Vicia faba* L.) is an annual diploid legume (2n = 2x = 12) and the seventh most produced legume worldwide with a yield of 3,503,300 tons in 2013 (FAOSTAT). It was one of the earliest domesticated legume species, with remains identified in Near Eastern sites as early as 10,700 BP [1]. It is a facultative cross-pollinating with outcrossing rates varying between 1–55% depending on environments [2]. It has a high protein content (2–25%) and is used for human and animal consumption. Faba bean is an important crop for sustainable agriculture in both marginal areas and advanced agricultural systems as it plays an important role in soil fertility and nitrogen fixation and it is able to grow in diverse climatic and soil conditions [1, 3, 4]. Narbon vetch (*V*. *narbonensis* L.) is another legume crop, which has some importance in the Mediterranean basin as it can cope with drought better than chickpea, faba bean and lentil [5]. *V*. *johannis* is a wild species of the *V*. *narbonensis* complex with agronomic interest for breeders due to increased frost tolerance and resistance to biotic stresses [6].

Faba bean gene pools have been defined based on seed size such as the major, minor and equina types [7]. The major type is common in the South Mediterranean countries and China; equina types are grown throughout the Middle East, North Africa and Australia and the small seeded, minor is found in North Europe [3]. Two main faba bean types have been proposed for Europe: a Central and Northwest European gene pool, consisting of *V*. *faba* var. minor and *V*. *faba* var. major types, and a Mediterranean gene pool which includes the former types but also *V*. *faba* var. equina [8].

The use of molecular markers has improved significantly the management and utilization of crop genetic diversity kept in germplasm collections [9–13]. In faba bean the large nuclear genome (13 Gb) hinders effective diversity characterization at the genomic levels, although some Single Sequence Repeat (SSR) and Single Nucleotide Polymorphism (SNP) markers have been developed [10–13]. Ninety-four Expression Sequence Tag-Single Sequence Repeats (EST-SSR) were used to analyze the genetic relationships among 32 genotypes, detecting distinct clusters based on geographical origins [12]. 657 SNPs used in 45 accessions of faba bean also permitted the detection of geographical clusters, with Mediterranean Basin and Near Eastern accessions clustering together but separately from Chinese accessions [4]. Genetic variability in floral biology, seed size, nutrient composition and stress tolerance has been identified among *V*. *faba* accessions [14]. To our knowledge, no studies addressed population structure in faba bean landraces in the Western Mediterranean scale, (including Atlantic islands) and compared these with wild related species.

The purpose of this work was 1) to assess the applicability of SSRs developed for *V*. *faba* as markers in the wild related species *V*. *johannis* and *V*. *narbonensis*; 2) to quantify genetic diversity in faba bean landrace accessions from the Western Mediterranean basin; 3) to identify population structure in faba bean landrace accessions from the Western Mediterranean basin.

## Materials and Methods

### Plant Materials

A panel consisting of 184 individual plants belonging to 53 *V*. *faba*, 2 *V*. *johannis* and 7 *V*. *narbonensis* accessions was assembled (S1 Table). Throughout this work, we refer to “accession” as a set of individual plants with the same provenance and stored in seed banks with the same ID. Cultivated *V*. *faba* accessions analyzed were originated from Portugal, Spain and Morocco (including the Atlantic Islands of Azores, Madeira and Canarias). Two accessions from Egypt and 2 from Ethiopia were included as outgroup. A commercial variety (5357) was also included. Accessions were provided by the Biotechnology and Genetic Resources Research Unit–Instituto Nacional Investigação Agrária e Veterinária (INIAV) Portugal, Centro de Recursos Fitogenéticos–Instituto Nacional de Investigación y Tecnología Agraria y Alimentaria (INIA) Spain and The Leibniz Institute of Plant Genetics and Crop Plant Research (IPK) Germany germplasm banks. Seeds were germinated in sterilized vermiculite and maintained in a climate chamber with controlled conditions (8 hours dark—15°C) / 16 hours light -22°C). DNA was extracted from young leaves of 3 individual plants of each accession using the Citogene® DNA Cell&Tissue Kit. (Citomed, Portugal).

### SSR genotyping

Twenty-eight faba bean SSRs were selected from the markers developed by Suresh, Park [15] to screen our accession panel. Primer sequences and details are described in S2 Table. Sequences of forward primers included an M13-tail at the 5’ end for attachment to a fluorescently-labelled M13 primer [16]; four fluorescent dyes were used: 6-FAM, PET, NED or VIC. PCRs were performed in a multiplex of 8 markers using a Multiplex PCR Kit (QIAGEN). Polymerase Chain Reaction (PCR) conditions were as described in Suresh, Park [15]. The DNA samples were organized in two 96-well plates with three different samples repeated in both as positive controls for scoring. All PCR amplification products were visualized on 1% tris/borate/ethylenediaminetetraacetic acid (TBE) -agarose gels stained with SYBR® Safe. SSR PCR products were separated by capillary electrophoresis on an automatic sequencer *ABI3130xl Genetic Analyzer* (AB Applied Biosystems). Fragments were scored against the GeneScan-500 LIZ Size Standard using the GENEMAPPER 4.1 (Applied Biosystems) and manually checked twice.

### Genetic Diversity

Allele frequencies and genetic diversity measures were calculated using PowerMarker 3.25 [17] and GenAlEx 6.5 [18]. These measures included number of alleles (Na), number of private alleles (Pa), genotype number, expected heterozygosity or Gene Diversity (H_E_), observed heterozygosity (H_O_), inbreeding coefficient (fixation index, F) and polymorphic information content (PIC). These measures were calculated for markers, accessions and categories (*ie*: wild vs cultivated; species; geographic provenance). Pairwise geographic distances between accessions, pairwise F_ST_ between accessions in the different groups and analysis of molecular variances (AMOVAs) were calculated using GenAlEx 6.5, with 999 permutations for testing variance components. To investigate Isolation-by-Distance we plotted pairwise F_ST_ and pairwise Nei’s Genetic Distance (*D*) measures against pairwise geographic distances.

### Population Structure

Population structure was assessed by three different methods: the Bayesian model-based approach implemented in the STRUCTURE v.2.3 software [19], Principal Component Analysis (PCA) and Neighbor-Joining (NJ) Phylogenetic Trees.

STRUCTURE was run with values of *K* ranging from 1 to 12, with 200,000 burn-in iterations and 1,000,000 Markov Chain Monte Carlo (MCMCs), with 10 independent runs for each K, using the admixture model with correlated allele frequencies. The most likely values of K were chosen based on *ΔK* according to the Evanno, Regnaut [20] method, computed with StructureHarvester [21]. STRUCTURE was run for three levels of accessions: the complete dataset including wild accessions, for the cultivated faba bean accessions only and for the Portuguese accessions only. Principal Component Analysis (PCA) was computed with the R environment for statistical computing using the package FactoMiner [22]. Like Structure, PCA was computed for three different levels of accessions. Computation of PCA was based on a matrix of allele frequencies for both individuals and accessions. Genetic distances *D* [23] between accessions and groups of accessions were calculated in PowerMarker with dendrograms constructed using a neighbor-joining clustering method with bootstrap support (1000 replicates) obtained by re-sampling the allelic frequency data. A majority-rule consensus tree was produced using the CONSENSE routine in the PHYLIP package available in the Mobyle portal (http://mobyle.pasteur.fr/cgi-bin/portal.py#welcome) and subsequently manipulated in FigTree v.1.4.2 [24].

## Results and Discussion

### SSR genotyping

Of the 28 markers tested, two did not produce any amplification (GBSSR-VF-21 and 271) and two were monomorphic across our accession panel (GBSSR-VF-34 and 276). For the successfully amplified SSRs the chromatograms were clear and markers easy to score. The results obtained for all markers and all accessions in our panel are detailed in S1 Table. A total of 104 alleles were detected in the 184 individual plants analyzed. The number of alleles detected per locus for the polymorphic markers ranged between 2 and 10 (GBSSR-VF-8) with an average of 4 alleles per locus (S3 Table). PICs ranged between 0.662 (GBSSR-VF-52) and 0.071 (GBSSR-VF-154) with a mean of 0.333 (S3 Table). For the markers tested, observed heterozygosity (*H*_*O*_) ranged from 0 (GBSSR-VF-34 and 276) to 0.467 (GBSSR-VF-52). These values are similar to the range between 0.000 and 0.500 reported by Gong, Xu [25] in a study of 11 SSRs in 29 accessions from China and Europe, but lower than the values observed in a trial of 150 SSRs in 32 worldwide accessions (0.091 to 0.841) [12].

Whereas 26 SSRs were successfully amplified in cultivated faba, only 16 worked in the wild accessions *V*. *johannis* and *V*. *narbonensis*. No differences in amplification success were observed between the two latter taxa. Differences are expected when markers developed for a particular species are tested in a different one that, although related, may have mutations in the SSRs flanking regions that prevent successful PCRs [26]. A similar hindrance in cross-species transferability was observed in other studies, as the following examples. Out of four retrotransposon-based Specific Sequence Amplification Polymorphism (SSAP) markers, three produced comparable results in both *V*. *faba* and *V*. *narbonensis*, but one of them was only effective in the latter [5]. Out of the 31 SSRs tested by Akash and Myers [10], only 10 amplified across different *Vicia* species and a few were specific of *V*. *faba* alone. The observed molecular behavior corroborates the classification *V*. *johannis* and *V*. *narbonensis* as distinct but still closely related species from *V*. *faba*. Moreover, this serves to show that SSRs developed in *V*. *faba* can be used to genotype wild accessions and other related cultivated species, although small-scale trials to test marker efficiency are recommended.

Since some markers only worked on cultivated materials, we subsequently analyzed the data on three levels: 1) the whole panel consisting of the three different species and the 16 SSRs that worked for all accessions; 2) the set of cultivated faba bean screened with 26 SSRs successfully amplified; 3) a geographic subset of accessions (in this case Portuguese accessions) for a small scale analysis.

### Genetic Diversity and its Distribution

Heterozygosity expected under Hardy-Weinberg equilibrium (*H*_*E*_) was relatively high (0.272) for the cultivated faba accessions but lower for the wild accessions (0.194) (Table 1). Among the wild taxa, *V*. *narbonensis* had a higher genetic diversity—measured as observed heterozygosity (*H*_*O*_)—than *V*. *johannis*. The differences in *H*_*O*_ between cultivated and wild accessions (0.204 and 0.017 respectively) were even more notorious. This could reflect a much more intense and widespread cultivation of *V*. *faba* as opposed to the small and spatially localized occurrence of *V*. *johannis* and *V*. *narbonensis*. In the first case it would create the opportunity for new variants to arise, whereas the fragmented and small-scale distribution of the wild populations would create bottlenecks and stronger genetic drift effects. Alternatively, the degree of inbreeding is much higher in *V*. *johannis* and *V*. *narbonensis* than it is in *V*. *faba* due to the predominantly self-pollination [27, 28].

**Table 1:** Summary of genetic diversity measures of accessions based on polymorphic SSRs.

Group		N	Na	Pa	H_o_	He	F
Biological type							
Cultivated	Mean (SE)	158	3.375 (0.507)	30	0.204 (0.041)	0.272 (0.047)	0.297 (0.066)
Wild	Mean (SE)	26	1.875 (0.287)	6	0.017 (0.011)	0.194 (0.061)	0.777 (0.092)
Taxon							
*V*.*faba*	Mean (SE)	158	3.375 (0.507)	30	0.204 (0.041)	0.272 (0.047)	0.297 (0.066)
*V*.*johannis*	Mean (SE)	6	1.125 (0.085)	0	0.010 (0.010)	0.041 (0.032)	0.455 (0.193)
*V*.*narbonensis*	Mean (SE)	20	1.688 (0.237)	3	0.017 (0.012)	0.170 (0.058)	0.731 (0.116)
Geographical provenance (cultivated accessions)							
Commercial	Mean (SE)	3	1.654 (0.156)	0	0.186 (0.057)	0.209 (0.045)	0.092 (0.125)
East	Mean (SE)	12	2.462 (0.249)	2	0.232 (0.043)	0.292 (0.044)	0.164 (0.070)
Morocco	Mean (SE)	8	2.385 (0.201)	4	0.264 (0.051)	0.326 (0.045)	0.186 (0.090)
Portugal	Mean (SE)	114	3.269 (0.370)	10	0.244 (0.039)	0.318 (0.046)	0.218 (0.042)
Spain	Mean (SE)	21	2.846 (0.270)	4	0.170 (0.032)	0.320 (0.040)	0.471 (0.070)
Region of Origin (Portuguese accessions)							
Azores	Mean (SE)	15	2.077 (0.207)	0	0.200 (0.038)	0.258 (0.041)	0.239 (0.052)
Centre	Mean (SE)	24	2.923 (0.318)	3	0.221 (0.038)	0.323 (0.046)	0.267 (0.060)
Madeira	Mean (SE)	33	2.615 (0.289)	1	0.268 (0.043)	0.300 (0.045)	0.081 (0.039)
North	Mean (SE)	18	2.385 (0.272)	1	0.208 (0.041)	0.288 (0.048)	0.282 (0.056)
South	Mean (SE)	24	2.808 (0.283)	6	0.289 (0.043)	0.341 (0.047)	0.110 (0.046)

Different categories: type (cultivated vs wild), taxon, geographical provenance (for cultivated accessions only) and region of origin (Portuguese accessions only). Standard errors (SE) of each measure are shown, if applicable.

***N***: sample size–number of individuals; ***Na***: number of alleles; ***Pa***: number of private alleles; **H**_**O**_: Observed Heterozygosity; **H**_**E**_: Expected Heterozygosity; ***F***: Fixation Index (Inbreeding Coefficient).

Within the cultivated types, Morocco harbored the highest diversity whereas Spanish accessions were the least diverse (Table 1). Considering that Spain is one of the major countries concerning germplasm diversity these results should be taken carefully. In our analysis we have just studied 7 *V*. *faba* Spanish accessions from different geographic location and these samples may not be a good representation of the global Spanish diversity.

Regional differences in genetic diversity were also observed at a country scale (Portuguese accessions) with the south of Portugal being the most diverse region. In all categories considered (Type, Taxon or Region), *H*_*O*_ was lower than *H*_*E*_ indicating that all faba bean groups are affected by some degree of inbreeding. Nevertheless, when each accession is analyzed individually (S4 Table), some have *H*_*O*_ values higher than *H*_*E*_ suggesting that outbreeding rates vary from accession to accession. Caution is advised, though, as we selected a small number of individuals per accession and as such this observation requires a future study with more individuals per accession.

Similarly, F was much higher in wild taxa than in the cultivated *V*. *faba*. An F value of 0 indicates perfect random mating in the population (*Hardy-Weinberg Equilibrium*) whereas values close to 1 are evidence of an excess of homozygosity (F = 1 means all individuals are homozygous) possible due to inbreeding. Negative values indicate outbreeding [29]. Although we had a small sample size for each accession (3 individuals), small sample sizes can still provide reliable estimations if the standard deviation is acceptable [30]. The higher values of F in the set of wild accessions suggest inbreeding is much higher than in the cultivated types. Likewise, a low F value was observed in the commercial variety 5357 (although the standard error computed was higher than the value itself), that means a high heterozygosity level for this variety. The lowest F value (0.081) was observed in the Madeira accessions, suggesting that new varieties were recently introduced or that outbreeding rates in these accessions are higher. The highest F value (0.471) occurred for the set of Spanish accessions suggesting that accessions from this region are more inbred. As with heterozygosity, when the data for each accession is considered there is wide variation in *F* values, suggesting that some accessions are predominantly outbreeding whereas others are mostly self-pollinating. These differences in the frequency of self-pollinating individuals are to be expected as *V*. *faba* is a facultative inbreeder. Field studies are necessary to confirm or falsify this hypothesis.

To test if differences in inbreeding coefficient could be related to climatic differences, we plotted *F* values against four climatic variables characterizing the locations where accessions came: average daily temperature (°C), average yearly rainfall (mm), average highest temperature (°C) and average lowest temperature (°C) (S1 Fig). None of these variables correlated highly with *F* (the highest *r*^*2*^ observed was 0.0569 for Average Highest Temperature). This means that the climatic variables tested do not visibly affect the accessions’ reproductive habit. Alternatively, microclimatic conditions could be in place at the particular locations where the accessions originated or maintenance in germplasm banks has affected *F* values. More likely, these differences in *F* values across different accessions could be attributed to differences in population size making inbreeding more frequent in smaller fields or to the introduction of different germplasm in historic times in particular locations.

**Fig 1:**
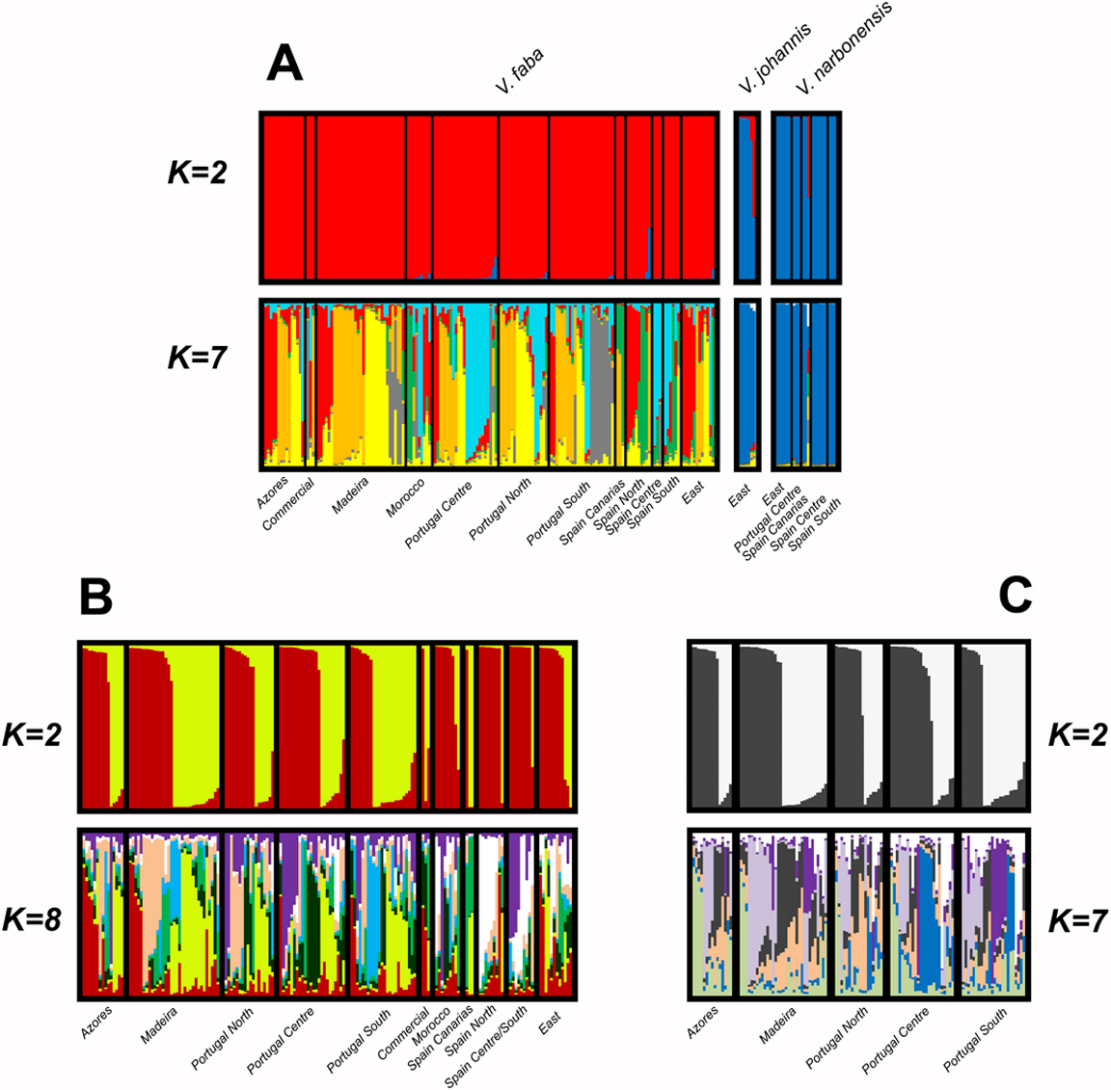
STRUCTURE analysis of all faba individual plants organized by species and regions. Clustering of faba individual plants based on multilocus analysis using the package STRUCTURE. Three levels of analysis are displayed **A)** all cultivated and wild accessions; **B)** cultivated accessions; **C)** Portuguese accessions. The two best fitting models according to Evanno’s *ΔK* are shown for each level. Accessions are organized by taxa and by region of provenance. Each individual is represented by a vertical line segmented into *K* colored sections. The length of each colored section is proportional to the membership coefficient (Q) of the individual accession to each one of the *K* clusters. Thin black vertical lines separate different regions.

An AMOVA analysis showed that for the cultivated types the highest proportion of genetic variability is found within accessions and not among different accessions or even among regions (Table 2). An AMOVA analysis based on Inter-simple sequence repeat (ISSR) marker data for 20 Greek faba landraces detected a much higher proportion of genetic variation within populations (75.4%) [8]. This is not too far out from our result of 85% when Portuguese accessions are considered or 81% for the set of cultivated accessions (Table 2). The high variability within accessions themselves was further evidenced when individuals from the same accession would fall clearly into different clusters in the *K =* 2 model of the STRUCTURE analysis (Fig 1), revealing that individuals from the same accession can in fact be genetically more similar to individuals from far way accessions than from individuals from the same population (*see section ‘Population Structure‘*). This surprisingly high heterogeneity of faba bean populations can be tentatively explained by the partially cross-pollinating reproductive habit of this species, for the mixture of hybrids from different sources and perhaps from a dynamic exchange of seed within neighboring farming communities. Many accessions of faba consist of a mixture of the phenotypically distinct *major*, *minor* and *equine* types, although sometimes these are cultivated separately [8].

**Table 2:** Analysis of molecular variance (AMOVA).

**Biological Type (Wild vs Domesticated)**				
	**df**	**SS**	**Est. Var.**	**%**
**Among Type**	1	249.584	2.745	54%
**Among Accessions**	60	282.423	0.487	10%
**Within Accessions**	304	556.417	1.830	36%
**Total**	365	1088.423	5.062	100%
**Taxa (Species)**				
	**df**	**SS**	**Est. Var.**	**%**
**Among Species**	2	274.033	2.888	56%
**Among Accessions**	59	257.974	0.431	8%
**Within Accessions**	304	556.417	1.830	36%
**Total**	365	1088.423	5.149	100%
**Regions of geographical provenance (cultivated accessions)**				
	**df**	**SS**	**Est. Var.**	**%**
**Among Regions**	4	55.260	0.152	3%
**Among Accessions**	48	404.321	0.752	16%
**Within Accessions**	263	1034.083	3.932	81%
**Total**	315	1493.665	4.837	100%
**Region of Origin (Portuguese accessions)**				
	**df**	**SS**	**Est. Var.**	**%**
**Among Regions**	4	40.996	0.057	1%
**Among Accessions**	33	254.013	0.622	13%
**Within Accessions**	190	753.333	3.965	85%
**Total**	227	1048.342	4.644	100%

AMOVA results for 184 individuals, 62 accessions, 3 taxa, 2 biological types, 5 geographic regions (for cultivated accessions only) and 5 regions of origin (Portuguese accessions only). F_st_ values and probability P(rand > = data) were as follows: Biological Type (0.638; 0.001), Taxa (0.645; 0.001), Regions of Geographical Provenance (0.187; 0.001), Region of Origin (0.146; 0.001).

Df: Degrees of Freedom; SS: Sum of squares; MS: Mean squares; Est. Var.: estimated variance; %: proportion of genetic variability

### Genetic Distance

To evaluate genetic differences between the accessions we computed F_ST_ and Genetic Distance *D* values between all pairs of accessions, for the three levels studied (all cultivated and wild accessions; cultivated accessions; Portuguese accessions). For the whole accession panel these values varied widely indicating that some accessions may be closely related among themselves than others. There were many pairs of cultivated accessions with an F_ST_ value of 0, which would indicate they interbreed freely and no reproductive barriers occur between them (panmixia). Some of these are geographically quite separated, for example accessions *BGE19747* and *5360* are from the Canary Islands and the Azores, respectively (S5 Table) but could descend recently from a common population. Nevertheless, it is significant that this low F_ST_ only occurs between cultivated faba materials and that all the wild accessions present high values in pairwise comparisons with the cultivated accessions and even amongst themselves (sheet 1 in S5 Table). The highest F_ST_ values (0.909) is between *V*. *narbonensis* accession *NAR139* from Spain and accession 5408, a *V*. *faba* from the north of Portugal. This value close to 1 indicates almost complete reproductive isolation and almost no sharing of genetic diversity. The same is true of pairwise *D*, with the wild accessions being more distant to the cultivated and closer among themselves. The highest genetic distance (1.346) is found between accessions *BGE011729* (*V*. *narbonensis* from the south of Spain) and *BGE19747* (*V*. *faba* from Tenerife, Canary Islands) (sheet 1 in S5 Table). When only cultivated accessions are considered the highest F_ST_ occurs between accessions *5334* (Lisbon, center Portugal) and *BGE19747* (Tenerife, Canary Islands). Again many accessions have F_ST_ values of 0 (sheet 2 in S5 Table). The highest genetic distance between two accessions is also between the same *BGE19747* accession and another accession from Lisbon, *5333* (sheet 2 in S5 Table). On a country scale, the highest F_ST_ is between accessions *2242* and *5361* (north and center of Portugal, respectively) and the highest *D* value is also between northern accession *2242* and southern accession *5397*. The closest genetic distance between any two Portuguese faba accession pairs accessions *5337* (Faro, south of Portugal) and *2285* (Madeira) (sheet 3 in S5 Table).

**Fig 2:**
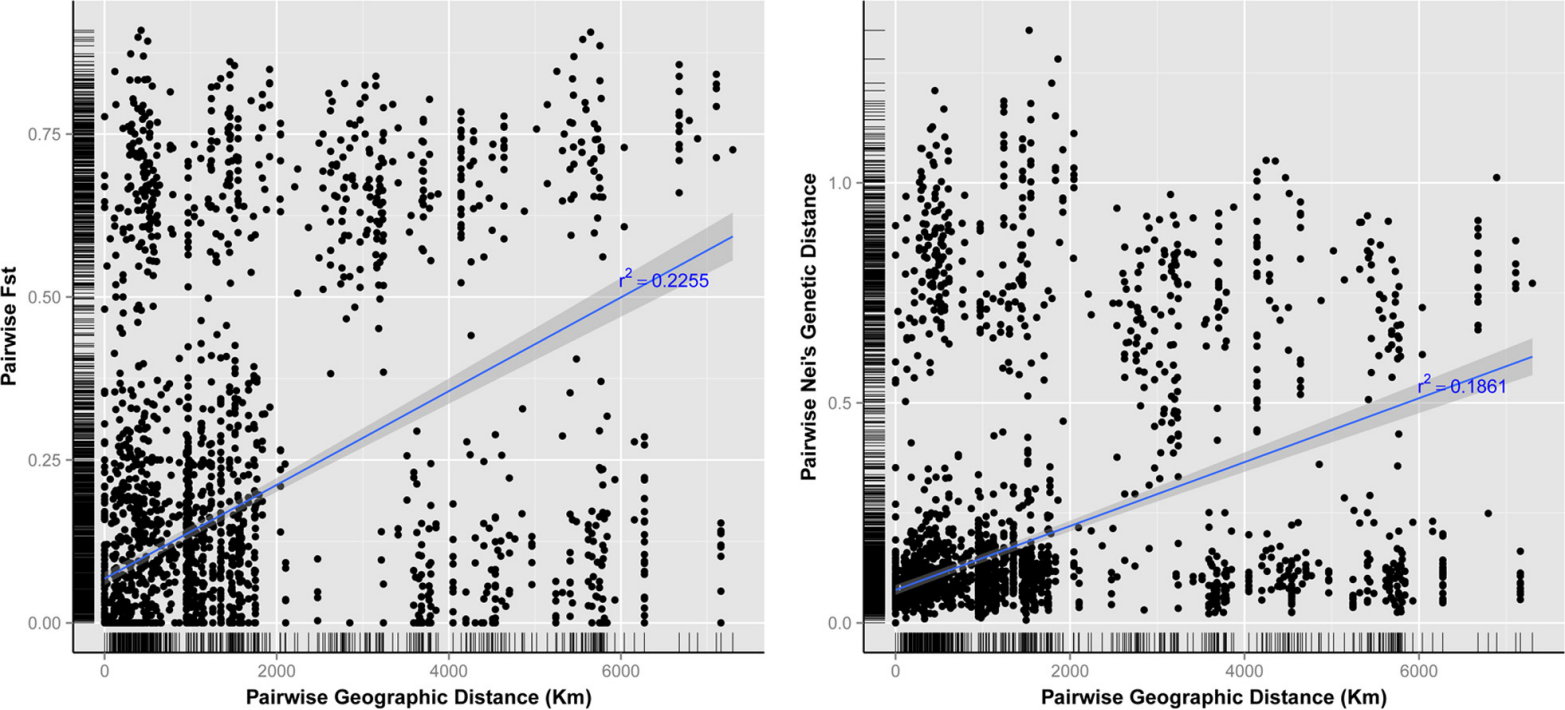
Isolation-By-Distance test for all cultivated and wild accessions. Testing Isolation-By-Distance (IBD) by plotting pairwise geographic distances against pairwise F_ST_ (left) and pairwise genetic distances (*D*) (right) for each pair of accessions genotyped in the all cultivated and wild accessions. Regression line and *r*^*2*^ values are shown in blue with the grey shading representing the 99% confidence region for the regression fit.

We tested if geographic distance between accessions could explain genetic differences (Isolation-by-Distance, or IBD). For that purpose we acquired the geographic coordinates for the place of origin of each accession in our panel and calculated the pairwise geographic distance between each pair of accessions in Km using the online tool available at http://biodiversityinformatics.amnh.org/open_source/gdmg/ (last consulted on 13^th^ of November 2015). We then harmonized and linearized the geographic distance pairwise matrix as well as the F_ST_ and *D* pairwise matrices (S5 Table) and plotted them, calculating the regression curve for each comparison. Again we did this for the complete set of accessions (Fig 2), for the cultivated accessions only and for the Portuguese accessions (S2 Fig). In all cases low correlations were observed for both F_ST_ and *D* and Geographic Distances, suggesting that genetic distances or differentiation is not a function of distance and that Isolation-By-Distance does not explain the genetic differences observed between accessions.

### Population Structure

In order to obtain information about population structure in faba bean accessions based on allele frequencies and not on any *a priori* classification such as provenance or taxonomy we used three methods: STRUCTURE, Principal Component Analysis (PCA) and Neighbour-Joining Phylogenetic Trees. As with other analysis we run STRUCTURE independently for the three different accession panels: all cultivated and wild accessions; cultivated accessions; Portuguese accessions. The computation of Evanno’s *ΔK* indicated *K = 2* as the most likely model for the three levels, but with *K =* 8 and *K =* 7 as the next most-likely models for the cultivated accessions and Portuguese accessions, respectively (S6 Table). For the whole panel *K =* 3 and *K =* 4 also had high *ΔK* values but we focused on the highest *K* model with an elevated *ΔK* in order to detect meaningful sub-populations (in this case *K* = 7). STRUCTURE results for the three levels are shown in Fig 1. At *K =* 2 with the complete set of accessions it is very clear that the cultivated *V*. *faba* represents a different gene pool (red in Fig 1A) from the wild *V*. *johannis* and *V*. *narbonensis* (blue in Fig 1A). No distinction seems to be detected between the latter two taxa. Moreover, at *K* = 7 cultivated faba bean is subdivided into different clusters whereas the wild taxa remain a uniform cluster. Both models indicate a very limited amount of gene flow between the wild and cultivated taxa.

In the cultivated faba bean runs, the *K =* 2 model indicates that each accession fits strongly into either of the two clusters. Accessions in Fig 1B were ordered by region of provenance and it is clear that in all regions both clusters are represented. When the Q-matrices are analyzed it is clear that even within accessions individuals receive alleles from different clusters. Nevertheless, when we calculated the genetic diversity for these two STRUCTURE defined clusters, the “yellow” cluster has a much higher genetic diversity and lower *F* values than the “red” cluster (*H*_*O*_ = 0.190, *H*_*E*_ = 0.285, *F* = 0.360 for the “red” cluster; *H*_*O*_ = 0.302, *H*_*E*_ = 0. 344, *F* = 0. 177 for the “yellow” cluster). This suggests that even within accessions some genotypes are more likely to interbreed than others. In the *K =* 8 model the gene flow and degree of admixture is rather visible with no particular clusters characterizing any single region (Fig 1B). The exceptions are the cluster painted white, that is present in the North and Centre/South of Spain but not anywhere else, and the light blue cluster more frequent in Portugal South and Madeira.

The same scenario is true for the runs with Portuguese accessions only: no cluster is found to be associated with any particular region (Fig 1C). Not even when individual Q-matrices for the Portugal accessions are plotted in a map for *K =* 2 and *K* = 7 (S3 Fig) is any particular geographic pattern discernible. Taken together, these results indicate that although the wild *Vicia* seems to be genetically quite distinct from the cultivated types, with very limited gene flow between them, there is no discrete geographic distribution of genetic diversity in the cultivated types. Even the cultivated accessions from as far as Egypt or Ethiopia do not constitute a genetically distinct cluster from the Western Mediterranean ones.

**Fig 3:**
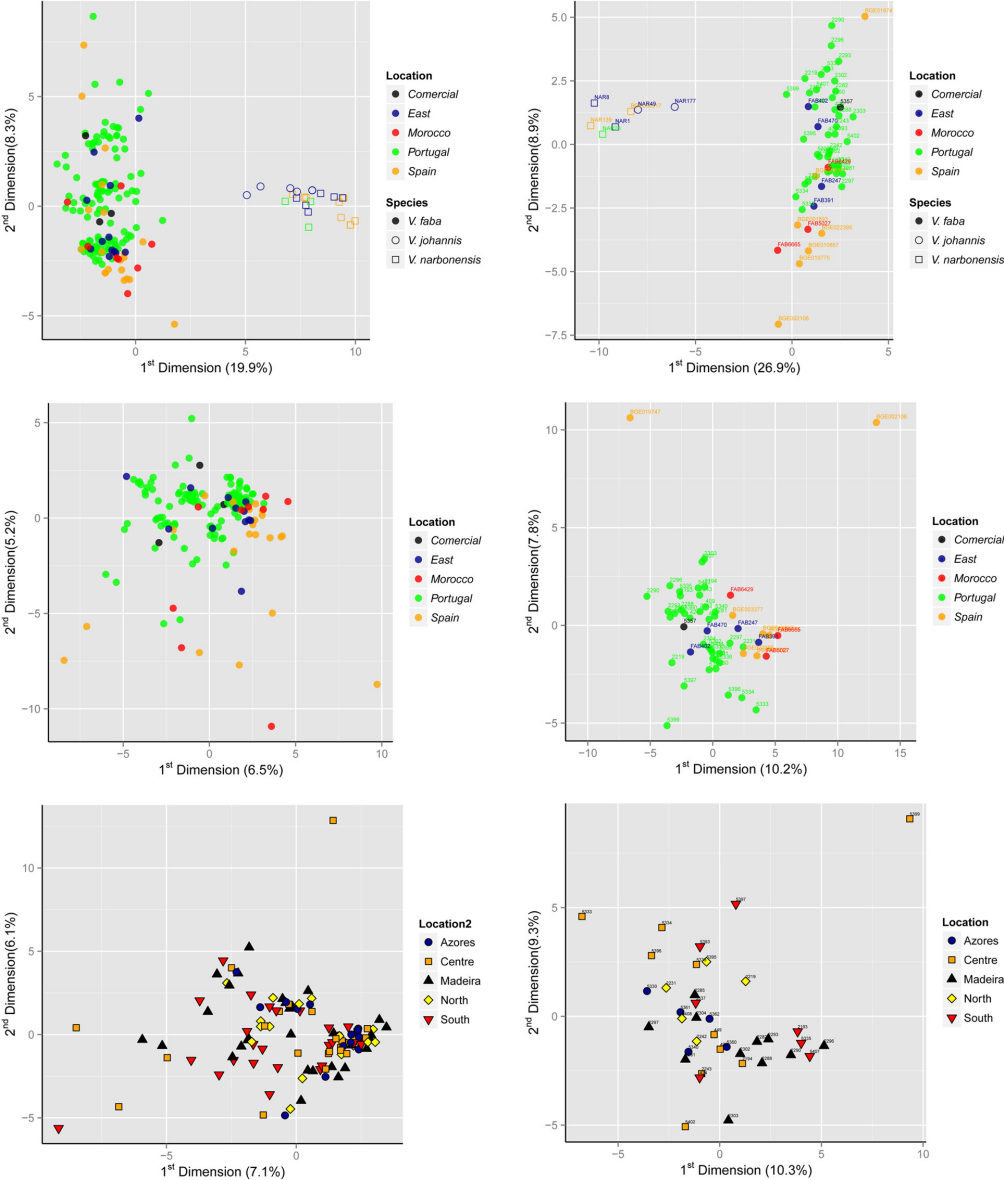
PCA analysis of allele frequencies from individuals and accessions. Plot of the 1^st^ and 2^nd^ components of a PCA analysis based on the individual (left panels) and accession (right panels) allele frequencies of polymorphic SSR markers. Each point represents an individual (left panels) or accession (right panels), with different symbols for the different taxa and each colored according to the region of provenance as described in its passport data. Three levels of analysis are displayed ***upper panel*)** all cultivated and wild accessions; ***middle panel*)** cultivated accessions; ***lower panel*)** Portuguese accessions. The proportion of variance explained by each component is given under brackets with each axis. The names of the accessions are provided with each point.

PCA corroborates STRUCTURE results. Separate PCAs was computed for individuals and accessions, with allele frequencies as input data. In both cases, when all cultivated and wild accessions are considered, it is visible that wild accessions are separated from the cloud of cultivated faba individuals (Fig 3, upper panel). Within the wild individuals and accessions, *V*. *johannis* is only tenuously separated from *V*. *narbonensis*. When only cultivated individuals and accessions are considered (Fig 3, middle panel) no clear geographic pattern is discerned, with the exception of two Spanish accessions (*BGE019747*, from the Canary Islands; *BGE002106* from Asturias, Spain) that seem to be quite distinct from all other cultivated accessions analyzed. Likewise, no clustering of points by region is observed when only the Portuguese individuals and accessions are considered (Fig 3, lower panel). No other distinct pattern is observed when other PCA components are plotted (S4 and S6 Figs).

**Fig 4:**
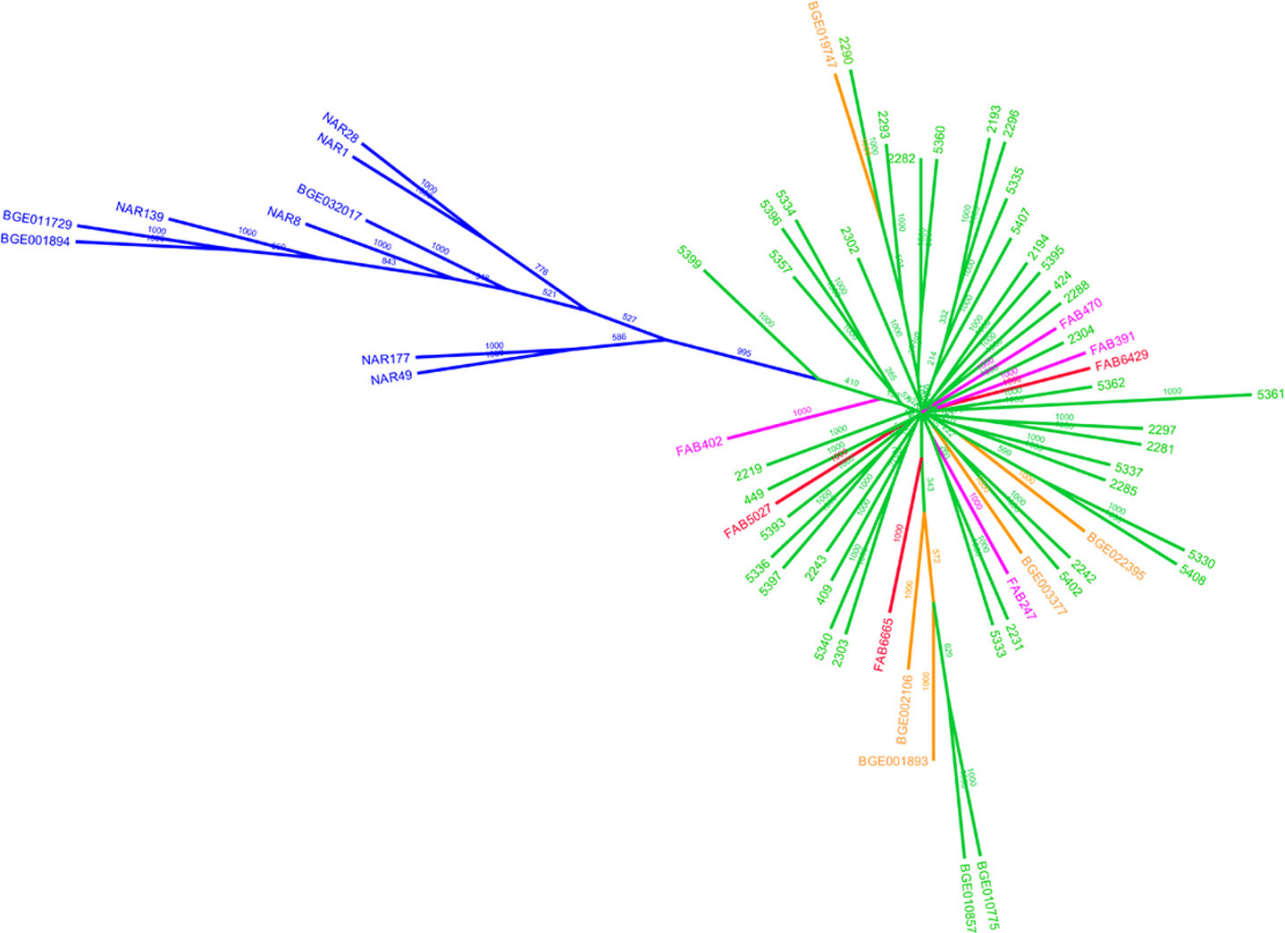
Consensus Neighbour-Joining tree for all wild and cultivated accessions. Consensus boot-strapped Neighbour-Joining tree of faba bean accessions based on the allele frequencies of polymorphic SSRs for all wild and cultivated accessions. The tree was constructed from Nei’s (*D*) genetic distances with 100 bootstrap replicates. The number of times the same node is retrieved in 1000 different trees is represented in each branch. The wild *V*. *narbonensis* and *V*. *johannis* accessions are coloured in blue; *V*. *faba* accessions form Portugal are shown in green, from Spain in orange, from Morocco in red and from the East (Egypt and Ethiopia) in purple.

The separation between wild and cultivated accessions is also detected in the NJ-tree based on genetic distances *D* (Fig 4). These are all clustered in a separate branch from all the cultivated types. Interestingly, the two *V*. *johannis* accessions (*NAR177* and *NAR49*) are placed in a branch apart from the one clustering the *V*. *narbonensis* accessions. Within the cultivated types, no branch seems to cluster accessions on the basis of a particular geographic provenance. We would expect accessions from the East (Egypt and Ethiopia) to be separated from the Portuguese and Spanish ones, due to the wide spatial range separating them, but no sort of is observed, the Eastern accessions are placed in different branches alongside Iberian accessions (Fig 4). The same is true for trees produced based on the cultivated accessions (S7 Fig) and on the Portugal accessions (S8 Fig). The high mixture and lack of genetic structure observed in our accessions using STRUCTURE and PCA suggests that the deducing relationships of genetic similarity or common descent from these trees are futile.

It is somehow intriguing not to detect population structure in *V*. *faba* when (rare) studies in other legumes are considered. Screening 45 retrotransposon-based markers in 3020 *Pisum* accessions, including landraces and wild materials, Jing, Vershinin [31] observed a clear structure based on taxonomy and, within the cultivated types, on geography. A geographical pattern in the distribution of the genetic diversity revealed by 21 SSRs in pea (*P*. *sativum* L.) landraces was also observed within China, corresponding to distinct regions in this country [32]. Sequencing of amplicons in 175 and 133 worldwide wild and cultivated lentil accessions respectively revealed a structure based on taxonomy and, within cultivated lentils, on seed size [33]. On a country scale (Ethiopia), the genotyping of 33 SSRs in chickpea identified five clusters distributed by geography [34]. Using 35 genomic and EST-SSRs, Blair, Diaz [35] distinguished major clusters within the American common bean (*Phasaeolus vulgaris* L.) and these were distributed according to geographic barriers. The same population structure was also detected using amplified fragment length polymorphisms (AFLPs) [36], SSRs [37] and SNPs [38]. Whole-genome re-sequencing, SNPs and SSRs also identified a geographical structure in Asian soybean accessions [39, 40].

More in accordance to our results, the screening of diversity arrays technology (DArT) marker in 300 accessions of chickpea identified three major clusters in the Mediterranean region, but accessions belonging to two distinct clusters were found co-existing in nearby regions in North Africa and Italy (Fig 1 in Thudi, Upadhyaya [41]). An absence of geographic patterning of population structure was also reported for the white lupin *Lupinus albus* L.) when 121 accessions were screened with AFLP markers [42]. Moreover, a recent study of 86 accessions from three species of genus Lens, using 12 genomic and 31 EST-SSR markers, although allowing the discriminations of several species revealed a higher genetic variability within accessions than among species or subspecies. [43].

This lack of geographic structure in the distribution of genetic diversity is also not uncommon in other cross-pollinated species, including crops. Genetic diversity in *Salix caprea* L. in Ireland is much higher within populations than among them and both chloroplast and nuclear SSR markers failed to detect a geographic population structure [44]. Both pollen-mediated and seed-mediated gene flow was high and the authors interpreted these results as due to human action through seed trade or transportation. Similarly, a screening of 15 nuclear SSRs in barley landraces across the whole of Ethiopia failed to detect any spatial based population structure, possibly due to high gene flow between regions [45]. A lack of correlation between genetic and geographic structure has been reported for cassava landraces in Mozambique [46] and sweet potato landraces in Brazil [47].

Zong, Redden [48] and Zong, Ren [49] used SSR to screen pea landraces from and outside China and AFLPs to screen Chinese faba bean landraces and compare these with a worldwide collection; in both cases they found that Chinese genotypes were distinct from the other accessions and found some structure within the former. Likewise, analyzing a collection of 151 worldwide faba bean accessions with 12 target-region amplification polymorphisms (TRAP), [50] Kwon et al were able to separate Chinese accessions from all other, with European accessions also forming a distinct cluster in a dendrogram. Similarly, a PCA of 79 Asian, European and North African faba bean accessions with AFLPs was able to distinguish Chinese accessions from all other with the Asian group partly separated from the others but the European and North African accessions from both *minor* and *major* groups were spread throughout without any particular grouping observed (Fig 4 in Zeid, Schon [51]). Screening retrotransposon based markers in *V*. *narbonensis* and *V*. *faba* accessions, Sanz, Gonzalez (5) detected only a weak structure and very little geographic clustering for both species, with a given geographical region often represented by multiple diverse groups. Likewise, using 17 SSRs in 43 accessions, Abid, Mingeot [52] found that Tunisian faba bean accessions formed a distinct cluster whereas accessions from other regions failed to cluster by geographic region. This is in agreement with our SSR results. Also in Tunisia, Yahia, Hannachi [53] found a correlation between SSR alleles and seed size, fitting the “*major*, *minor*, *equina”* classification. Likewise, Terzopoulos and Bebeli (8)] could see a separation of *minor* types from other faba bean types in a PCA plot based on the allele frequency data for four ISSR markers. A similar type of marker system was able to separate *V*. *faba* var. *major* from *V*. *faba* var. *minor* in Egypt [54]. Tomás, Silva [55] demonstrated the discrimination between faba bean´s *major* and *equina* groups based on seed traits analysis and inter retrotransposon amplified polymorphism (IRAP) markers. Contrastingly, retro-transposon based SSAP markers clearly separated *V*. *narbonensis* from *V*. *faba* but failed to cluster accessions on the basis of geographical origin or the *major-minor-equina* classification (Fig 2 in Sanz, Gonzalez (5)]).

At the beginning of the project we expected to be able to deduce from which regions in the mainland faba bean germplasm was introduced in Madeira and the Azores Islands. Alas, the lack of population structure in the cultivated germplasm and the high degree of admixture suggested by AMOVA and STRUCTURE prevents us from inferring any route of introduction. The only theory we could postulate is that germplasm from many different regions contributed to the insular gene pool. More likely, the first colons of those islands (15^th^ and 16^th^ centuries) brought the faba accessions from the regions where they departed, but these already harbored a very high genetic diversity. 400 years of isolation might have not been enough time for genetic drift and selection to create the population structure necessary to distinguish insular and mainland accessions. Alternatively, there has been a more or less constant influx of new faba accessions from the mainland, freely interbreeding with previously introduced varieties.

The lack of a population structure in our faba panel could be due to the fact that the SSRs we selected are not genomic but located in expressed regions of the genome (EST-SSRs), and hence less likely to accumulate mutations than markers located in neutral regions. Nevertheless, previous studies compared the performance of genomic and EST-SSRs in estimating genetic diversity, F_ST_, and population assignment and although genomic SSRs were slightly more diverse, both systems were found to be equally effective [55, 56]. We cannot exclude the possibility that a higher number of SSRs or a different genetic marker system (SNPs) would eventually detect population structure even at a regional level. Most likely, though, this absence of structure reflects the high degree of gene flow caused by the facultative outbreeding habit and the introduction of novel genotypes throughout the history of this species cultivation. For example, faba bean remains found in the archaeological record in the Mediterranean area from the Neolithic up to the Roman Period are almost always of the *minor* type [1]. The *major* type that now is commonly found everywhere in Europe probably only evolved around 500 AD, when other major changes in crop choices and agricultural practices were occurring in Europe such as a substitution of wheat for rye [56, 57]. This little time since the introduction of new varieties phenotypically of the *major* type may not have been enough for bottlenecks, isolation-by-distance, selection and genetic drift to differentiate between the accessions from different regions, especially when the effects of such dynamics are offset by the gene flow of cross-pollination and seed exchange.

## Conclusions

We were able to screen cultivated faba bean and wild related species with a set of SSRs developed for *V*. *faba*. Not all SSRs that produced results in the cultivated types worked in the wild *V*. *johannis* and *V*. *narbonensis* although the majority did so (16 out of 26). This shows that genetic markers developed for *V*. *faba* can be used in studies of wild related species. Our data also suggest that *V*. *johannis* and *V*. *narbonensis* are rather distinct taxa from *V*. *faba* and that limited gene flow existed among these taxa. A small degree of separation between the two wild taxa *V*. *johannis* and *V*. *narbonensis* could be observed. Genetic diversity analysis revealed that cultivated faba bean is more diverse than wild relatives and that within the latter *V*. *narbonensis* presents higher diversity than *V*. *johannis*. Similar levels of genetic diversity are found throughout the regions defined although the Spanish accessions have lower diversity and higher levels of inbreeding. The degree of outbreeding, as detected by computation of *F* values, varies among accessions and regions, but does not seem to be associated with climatic factors. The largest proportion of genetic variability is found within accessions, and not among accessions or even among accessions of different regions.

Apart from the separation between wild and cultivated accessions we could not detect any population structure based on geography in faba accessions from the Western Mediterranean region. Two major clusters occur within cultivated faba but both of them are widespread and can be found even within the same accessions. Not even the accessions from Egypt and Ethiopia used as outgroups appeared as different clusters.

Altogether, these results indicate that faba accessions harbor the majority of diversity found in this species and that a significant amount of outbreeding and gene flow (human-mediated movement of varieties or otherwise) exists among cultivars all throughout the range of this crop. Each local landrace seems to have its own evolutionary dynamics probably due to its facultative outbreeding habit. This prevents any meaningful structure to be evidenced by a small number of markers.

## Supporting Information

S1 Fig Correlations between inbreeding coefficient (F) for the cultivated accessions and four climatic variables. Clockwise from upper left: average daily temperature (°C), average yearly rainfall (mm), average highest temperature (°C) and average lowest temperature (°C). Regression line and *r*^*2*^ values are shown in blue.
(TIF)

S2 Fig Isolation by distance test for all cultivated, and only Portuguese accessions. Testing Isolation-By-Distance (IBD) by plotting pairwise geographic distances against pairwise F_ST_ (left) and pairwise genetic distances (*D*) (right) for each pair of accessions genotyped in the cultivated panel only (**A-B**) and the Portugal only accessions (**C-D**). Regression line and r2 values are shown in blue with the grey shading representing the 99% confidence region for the regression fit.
(TIF)

S3 Fig Geographical distribution of population structure in Portuguese individual. Geographical distribution of population structure in Portuguese individual faba plants according to the models *K =* 2 (upper map) and *K* = 7 (lower map) produced by STRUCTURE. Each individual is depicted as a pie chart with the proportional membership of its alleles to each one of the two (upper map) or seven (lower map) groups.
(TIF)

S4 Fig PCA analysis of allele frequencies for the all wild and cultivated faba bean accessions. Lettuce Plot of the 1^st^ to 4^th^ components of a PCA analysis based on the accession allele frequencies of polymorphic SSR markers for the all wild and cultivated faba beanaccessions. See **Fig 2** for legend.
(TIF)

S5 Fig PCA analysis of allele frequencies for the all cultivated faba bean accessions. Lettuce Plot of the 1^st^ to 5^th^ components of a PCA analysis based on the accession allele frequencies of polymorphic SSR markers for the cultivated faba bean accessions. See **Fig 2** for legend.
(TIF)

S6 Fig PCA analysis of allele frequencies for the Portuguese faba bean accessions. Lettuce Plot of the 1^st^ to 5^th^ components of a PCA analysis based on the accession allele frequencies of polymorphic SSR markers for the Portuguese faba bean accessions. See **Fig 2** for legend.
(TIF)

S7 Fig Consensus Neighbour-Joining tree for cultivated accessions. Consensus boot-strapped Neighbour-Joining tree between faba bean accessions based on the allele frequencies of polymorphic SSRs for cultivated accessions. The tree was constructed from Nei’s (*D*) genetic distances with 100 bootstrap replicates. The number of times the same node is retrieved in 100 different trees is represented in each branch.
(PDF)

S8 Fig Consensus Neighbour-Joining tree for Portuguese accessions. Consensus boot-strapped Neighbour-Joining tree between faba bean accessions based on the allele frequencies of polymorphic SSRs for Portuguese accessions. The tree was constructed from Nei’s (*D*) genetic distances with 100 bootstrap replicates. The number of times the same node is retrieved in 100 different trees is represented in each branch.
(PDF)

S1 Table List of individuals analysed and their alleles for each SSR. (XLSX)

S2 Table SSR markers used in this study, including primer sequences and expected sizes. (XLSX)

S3 Table Genetic diversity measures for the SSR markers screened in the three different levels of analysis. (XLSX)

S4 Table Genetic diversity measures for the accessions genotyped in the three different levels of analysis. Included are information about accessions (species, geographic coordinates, country provenance, type, region of origin).
(XLSX)

S5 Table Pairwise F_ST_ and pairwise genetic distance matrices for each pair of accessions. Each spreadsheet represents one of the three different levels of analysis: all cultivated and wild accessions; cultivated accessions; Portuguese accessions
(XLSX)

S6 Table Evanno’s *ΔK* calculations for the STRUCTURE runs with different accession panels. (XLSX)
