# Supplementary figures and images for: Genetic Diversity and Population Structure in Vicia faba L. Landraces and Wild Related Species Assessed by Nuclear SSRs
Source: PLoS One. 2016 May 11;11(5):e0154801. doi: 10.1371/journal.pone.0154801 (PMC4864303; doi:10.1371/journal.pone.0154801)

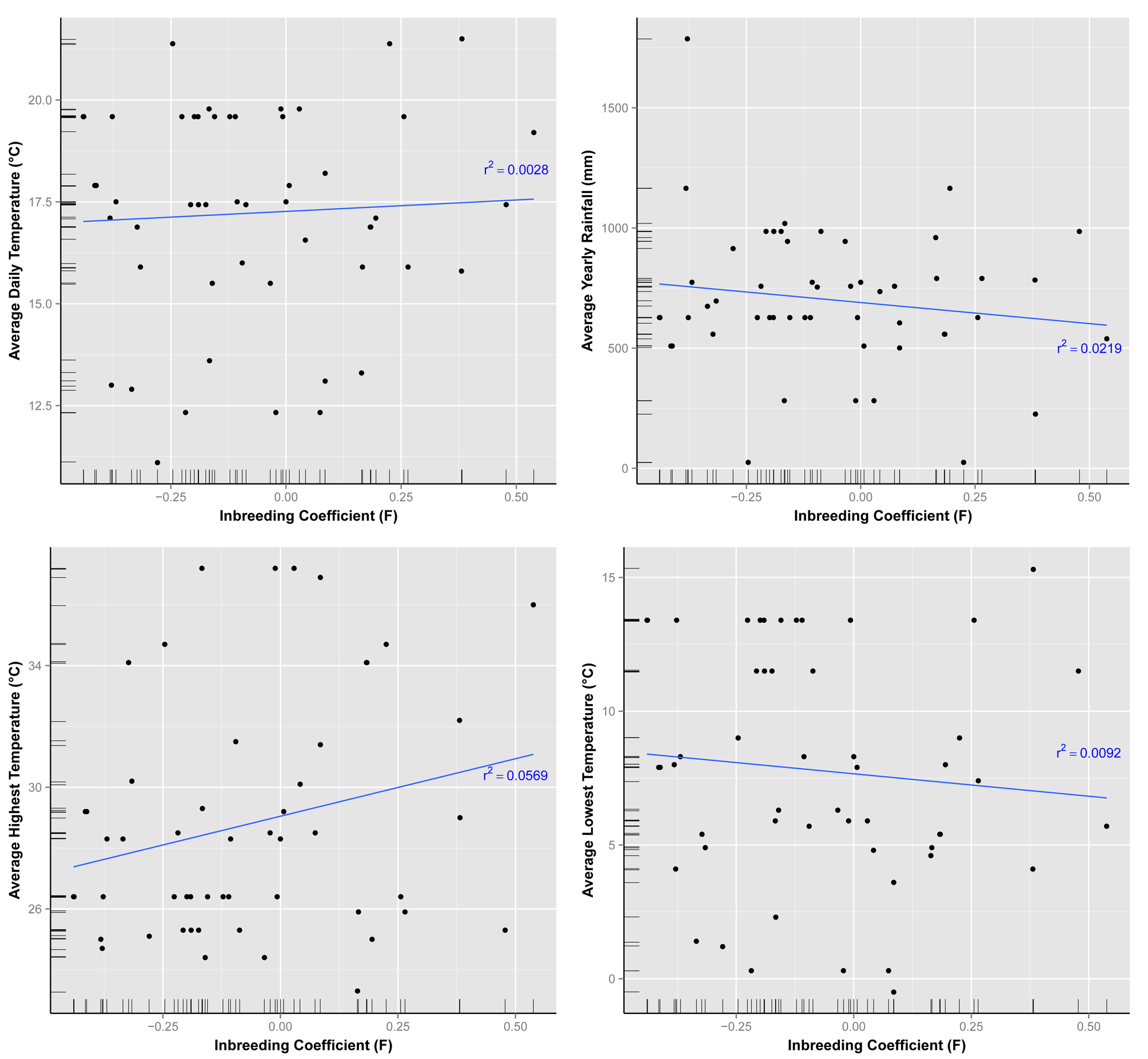

Supplement: S1 Fig — Clockwise from upper left: average daily temperature (°C), average yearly rainfall (mm), average highest temperature (°C) and average lowest temperature (°C). Regression line and r2 values are shown in blue. (TIF) [file pone.0154801.s001.tif]

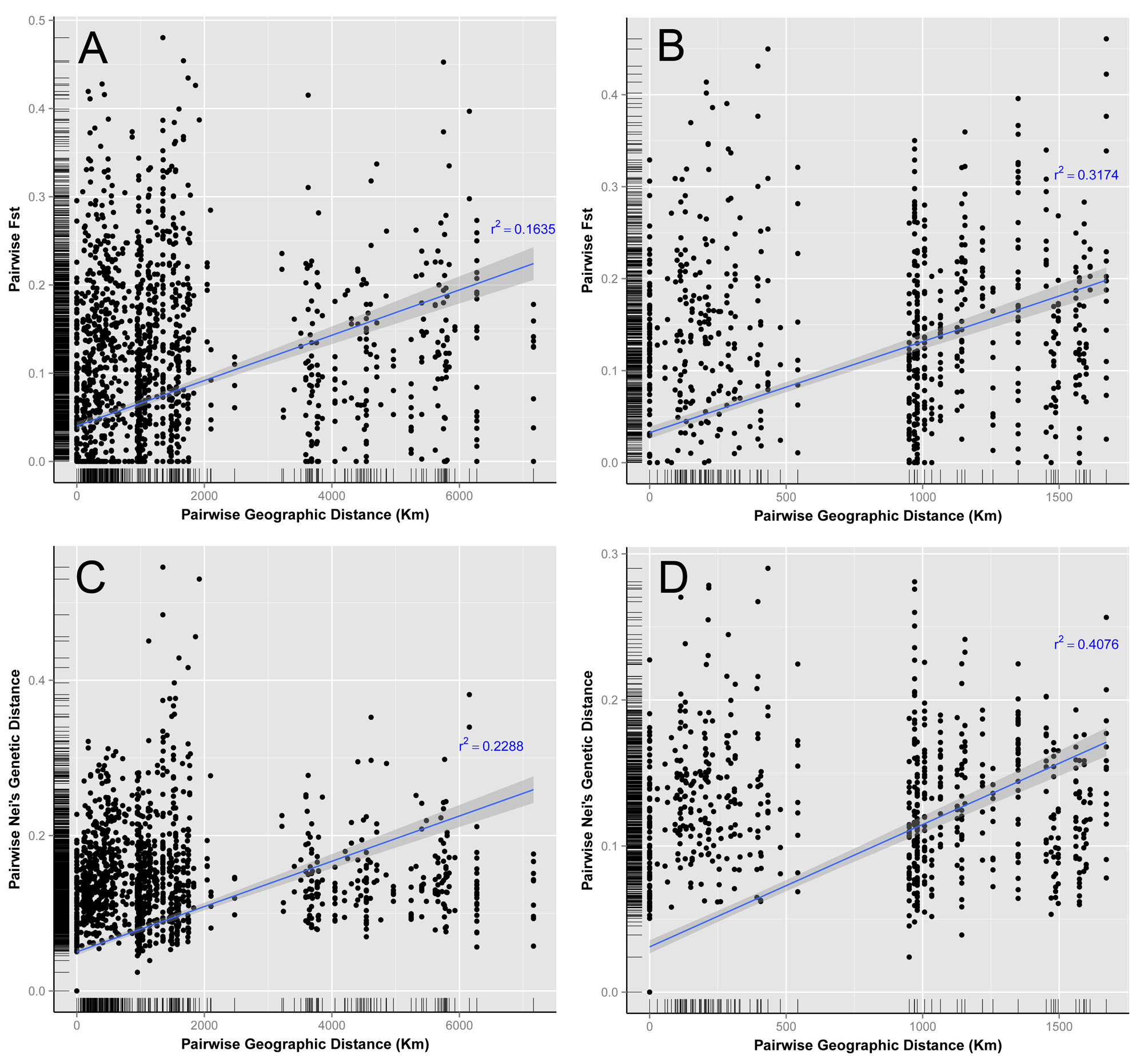

Supplement: S2 Fig — Testing Isolation-By-Distance (IBD) by plotting pairwise geographic distances against pairwise FST (left) and pairwise genetic distances (D) (right) for each pair of accessions genotyped in the cultivated panel only (A-B) and the Portugal only accessions (C-D). Regression line and r2 values are shown in blue with the grey shading representing the 99% confidence region for the regression fit. (TIF) [file pone.0154801.s002.tif]

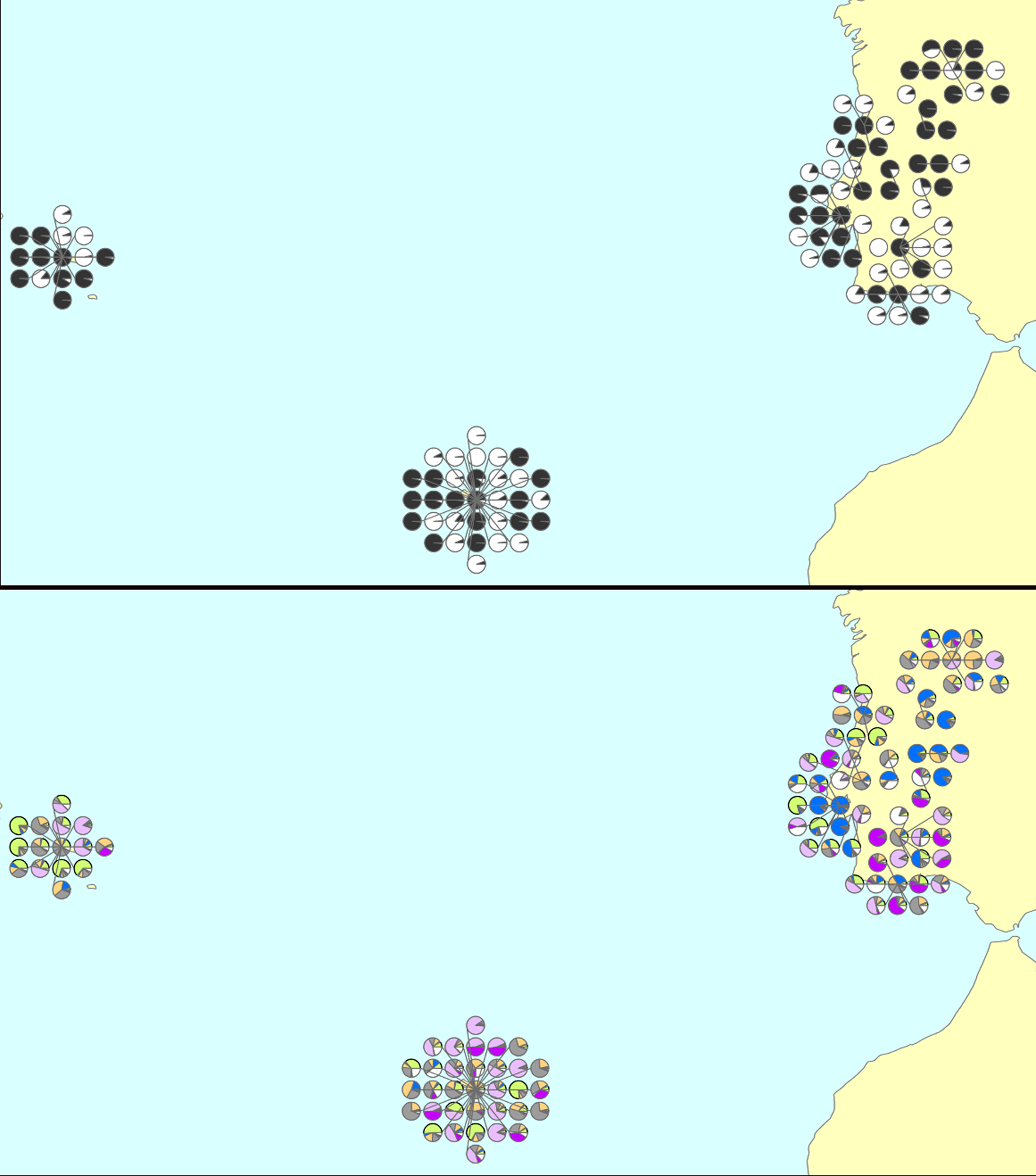

Supplement: S3 Fig — Geographical distribution of population structure in Portuguese individual faba plants according to the models K = 2 (upper map) and K = 7 (lower map) produced by STRUCTURE. Each individual is depicted as a pie chart with the proportional membership of its alleles to each one of the two (upper map) or seven (lower map) groups. (TIF) [file pone.0154801.s003.tif]

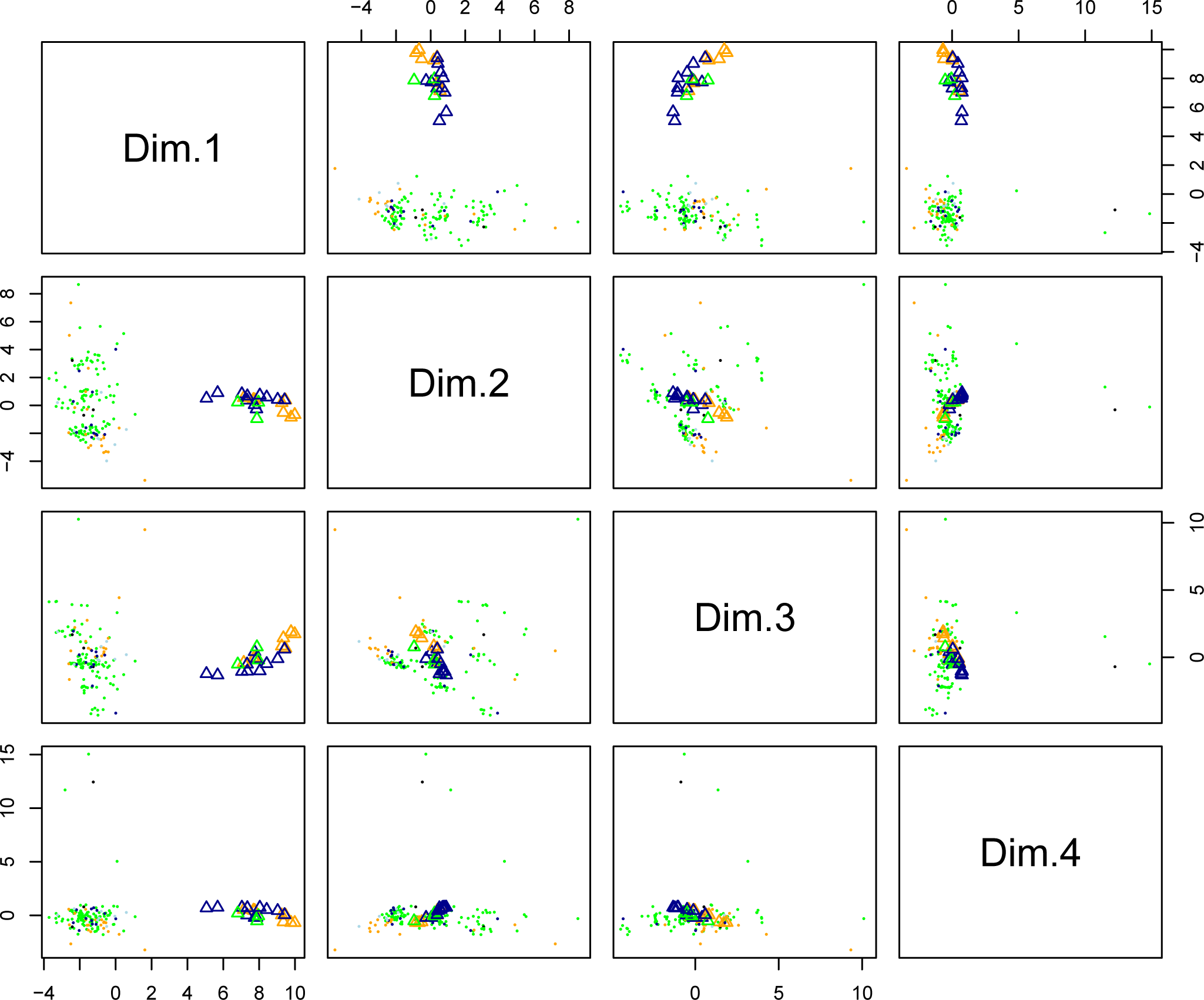

Supplement: S4 Fig — Lettuce Plot of the 1st to 4th components of a PCA analysis based on the accession allele frequencies of polymorphic SSR markers for the all wild and cultivated faba beanaccessions. See Fig 2 for legend. (TIF) [file pone.0154801.s004.tif]

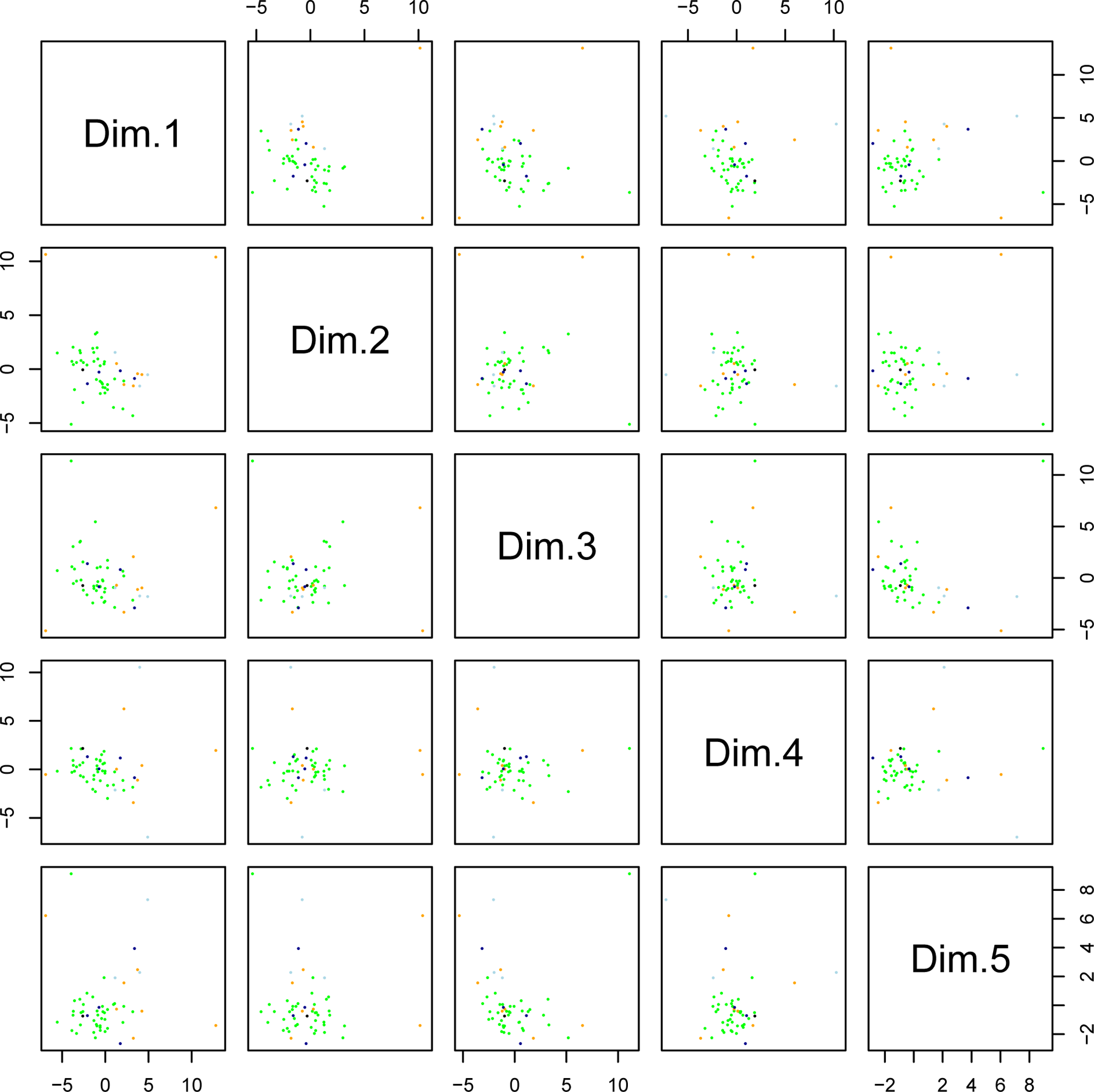

Supplement: S5 Fig — Lettuce Plot of the 1st to 5th components of a PCA analysis based on the accession allele frequencies of polymorphic SSR markers for the cultivated faba bean accessions. See Fig 2 for legend. (TIF) [file pone.0154801.s005.tif]

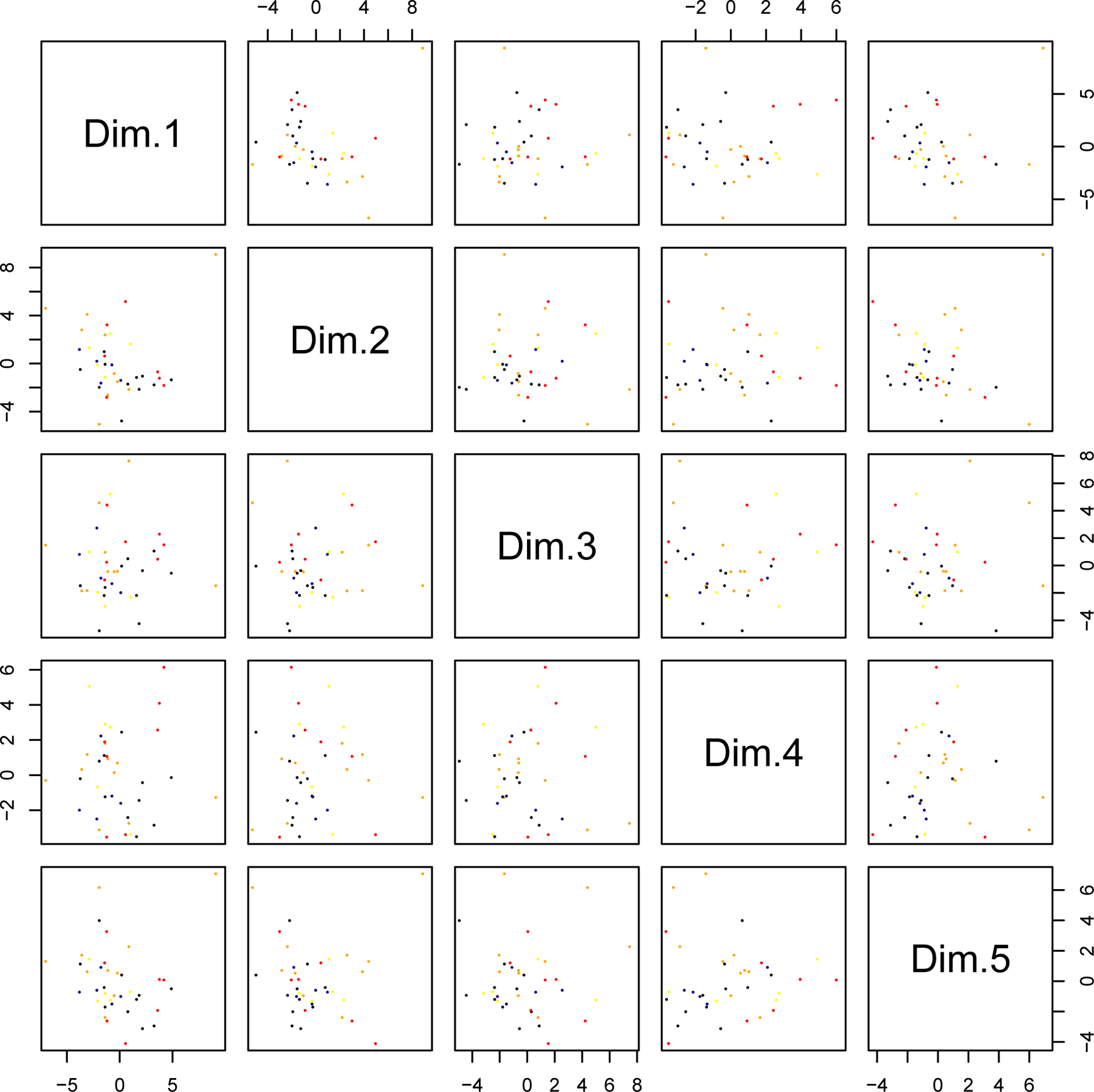

Supplement: S6 Fig — Lettuce Plot of the 1st to 5th components of a PCA analysis based on the accession allele frequencies of polymorphic SSR markers for the Portuguese faba bean accessions. See Fig 2 for legend. (TIF) [file pone.0154801.s006.tif]

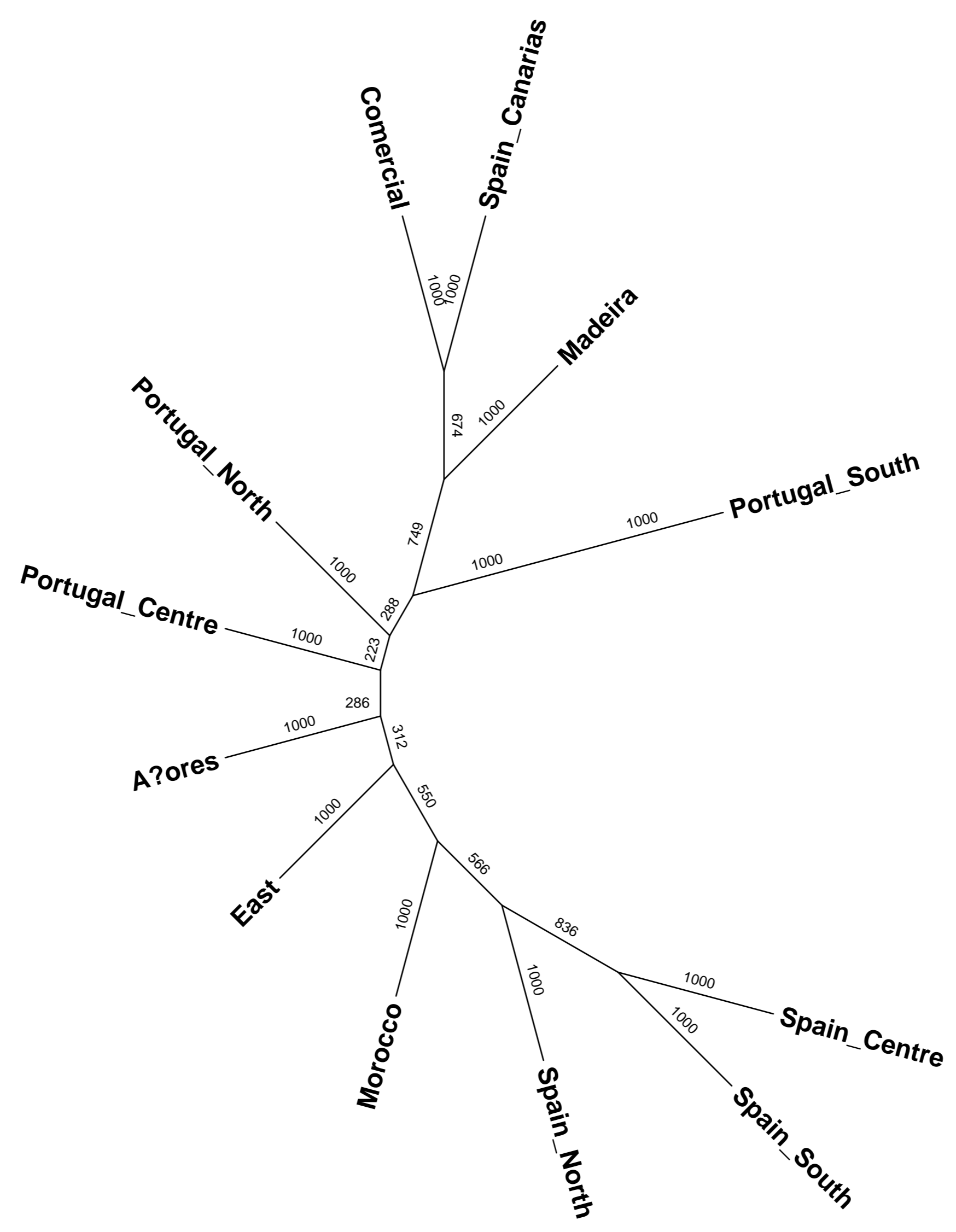

Supplement: S7 Fig — Consensus boot-strapped Neighbour-Joining tree between faba bean accessions based on the allele frequencies of polymorphic SSRs for cultivated accessions. The tree was constructed from Nei’s (D) genetic distances with 100 bootstrap replicates. The number of times the same node is retrieved in 100 different trees is represented in each branch. (PDF) [file pone.0154801.s007.pdf]

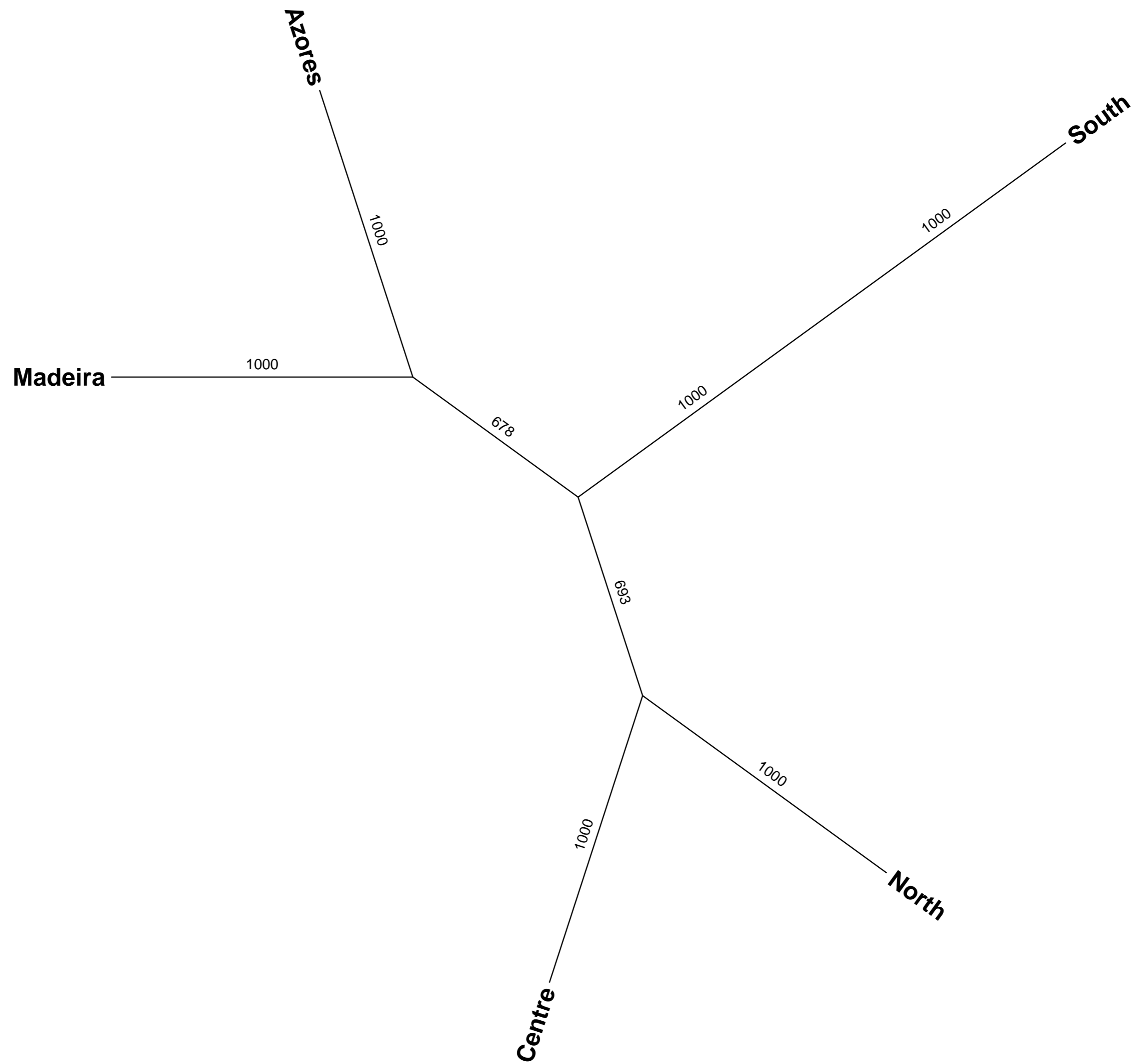

Supplement: S8 Fig — Consensus boot-strapped Neighbour-Joining tree between faba bean accessions based on the allele frequencies of polymorphic SSRs for Portuguese accessions. The tree was constructed from Nei’s (D) genetic distances with 100 bootstrap replicates. The number of times the same node is retrieved in 100 different trees is represented in each branch. (PDF) [file pone.0154801.s008.pdf]
